# Patient experiences, attitudes and expectations towards receiving information about anti-TNF medication: a quantitative study

**DOI:** 10.1007/s10067-017-3642-5

**Published:** 2017-05-19

**Authors:** Jon Packham, Paul Arkell, Tom Sheeran, Ann Brownfield, Anthony Cadwgan, Sarah Ryan

**Affiliations:** 1 Haywood Academic Rheumatology Centre, Stoke-on-Trent, UK; 2 Institute of Applied Clinical Science, Keele University, Keele, UK; 3 Cannock Hospital, Cannock, UK; 4 University Hospital of North Midlands NHS Trust, Stoke-on-Trent, UK

**Keywords:** Patient education, Rheumatoid arthritis

## Abstract

The objective of the study was to measure patient attitudes and experience of information received during drug counselling for rheumatoid arthritis (RA) medications. This is a cross-sectional UK postal questionnaire study. Three RA patient groups—disease-modifying antirheumatic drugs (DMARDs) only, first anti-tumour necrosis factor (anti-TNF) and failed anti-TNF—were sent postal questionnaires. Data on patient history/demographics, drug counselling experience, knowledge of drug side effects, attitudes to vaccinations, cancer screening and blood borne virus testing was collected; 264/679 (39%) patients responded (median age 65 years, 66% female, median disease duration 15 years). Drug information from rheumatology nurses, rheumatology doctors and information leaflets was most useful. Thirty-eight percent of respondents felt reassured by information received, but 37% felt more worried. Forty percent of participants were aware of important drug side effects. Although 42–65% of patients understood they should temporarily halt anti-TNF therapy with concurrent infection, 75% of patients recalled continuing therapy despite infection. Thirteen percent believed that all vaccinations (including travel vaccinations) were safe while taking anti-TNF. Uptake of UK cancer screening programmes was between 87 and 94%, except prostate screening (47%). Most participants were not aware that they may need to discontinue their anti-TNF if they developed cancer. The majority of participants felt neutral/reassured by the prospect of viral hepatitis (95%) and HIV (91%) testing. Although drug counselling is a well-established part of clinical care, there is potential for further improvement to ensure that patients’ knowledge empowers them to act safely. Particular areas for improvement included the following: patients halting DMARDs/anti-TNF therapy during infections, knowledge regarding vaccinations and prostate cancer screening uptake.

## Introduction

Patient education has an important role in the management of rheumatoid arthritis (RA). Patient education programmes can benefit patient’s disease knowledge and disease activity [1, 2]. Many rheumatology centres have both disease and medication educational programmes, with information from various sources (physicians, allied health professionals, patient support groups, other patients, family, internet/media) [3]. Increasingly, counselling is being provided by community-based health professionals, rather than hospital staff. Despite participation in drug counselling, patients often lack key knowledge about their disease/medication [4, 5].

Qualitative studies exploring attitudes towards RA patient education and drug counselling [6–11] identify high patient expectations of information provision, with general satisfaction regarding quality and timing; however, quantitative data are lacking. Increasing numbers of RA patients receive parenteral therapies [12, 13], with potentially serious side effects, posing new challenges in RA patient education [14, 15].

This is the first quantitative study, describing knowledge and attitudes about RA therapies from the patients’ perspective in the UK. Data has been particularly lacking on issues such as knowledge and uptake of vaccinations, cancer screening and acceptability of testing for blood-borne viruses. This study measured patient attitudes and experience of information received while starting immunosuppressive medications, with a particular focus on anti-TNF medications.

## Methods

This cross-sectional postal questionnaire study was conducted across a population of 807,000, 96% white Caucasians in Staffordshire, UK, requiring specialist rheumatology care for 5000+ RA patients, with over 1000 patients having received at least one biological agent. Ethical approval was obtained from the Staffordshire Local Research Ethics Committees. Written consent was obtained according to the Declaration of Helsinki.

Patients starting new therapy receive face-to-face drug counselling from a specialist nurse and written information on treatment and potential side effects. Those starting biologic therapy are assessed for malignancy and are screened for tuberculosis/hepatitis. Six hundred seventy-nine prospective participants were sampled from three existing lists of RA patients, using random number generation: group I: patients taking their first anti-TNF agent, group II: patients who had failed their first anti-TNF agent and group III: patients taking disease-modifying antirheumatic drugs (DMARDs) without exposure to biologic agents.

Data were collected by self-administered postal questionnaires, developed from a previous study [6] including patient history/demographics, drug counselling experience, knowledge of drug side effects and attitudes to vaccinations, cancer screening and blood-borne virus testing. Patient global assessment [16, 17] was used as an indication of RA disease impact and index of multiple deprivation based on postcode [18] -measured socioeconomic status.

Data were analysed with simple descriptive statistics: mean/standard deviation for continuous data and counts/proportions for categorical data. Associations between participant variables and responses used chi-square (two categorical variables), unpaired *t* (one categorical and one continuous variable) and Pearson correlation (two continuous variables) tests.

Knowledge about drug side effects were classified as incorrect if the anti-TNF groups did not recognise that anti-TNF could cause a rash, increased risk of infection and theoretical increased risk from cancer. DMARD participant responses were classified as correct if they recognised common potential side effects of DMARDS including rash, high blood pressure (leflunomide), abnormal liver blood tests (methotrexate, sulphasalazine and azathioprine), abnormal kidney blood tests (gold) and increased risk of infection (methotrexate and azathioprine).

Participants were only included in cancer screening analysis if they were eligible for screening based on age/gender [19–21]: bowel cancer screening participants aged 60–69 years, breast cancer (mammography) for women aged 50–70 years, cervical cancer (smear) for women aged 25–64 years, and prostate cancer (prostate-specific antigen (PSA)) for men aged over 50 years.

## Results

264/679 (39%) patients responded, with similar rates between patient groups. Sixty-six percent were female with median (IQR) age 65 (55–71) years, median (IQR) years since diagnosis 15 (8–25), and mean (SD) patient global assessment (PGA) 43 (27)/100. Demographic and clinical details for each patient group are shown in Table 1.

**Table 1:** Demographic and clinical information for each patient group

	First anti-TNF patients (*n* = 95)	First anti-TNF failures (*n* = 84)	DMARD patients (*n* = 80)
Female *n* (%)	64 (66)	61 (72)	49 (61)
Median (IQR) age	64 (52–72)	65 (56–71)	65 (57–72)
Patient global assessment—VAS score, mm (SD)	39 (24)	53 (28)	39 (28)
Infliximab *n* (%)	23 (26)	23 (35)	–
Etanercept *n* (%)	24 (27)	17 (26)	–
Adalimumab *n* (%)	31 (35)	21 (32)	–
Certolizumab *n* (%)	11 (12)	4 (6)	–
Single therapy *n* (%)	–	–	44 (55)
Dual therapy *n* (%)	–	–	27 (34)
Triple therapy *n* (%)	–	–	9 (11)
Methotrexate *n* (%)	–	–	60 (75)
Sulphasalazine *n* (%)	–	–	27 (34)
Leflunomide *n* (%)	–	–	11 (14)
Hydroxychloroquine *n* (%)	–	–	23 (29)
Azathioprine *n* (%)	–	–	3 (4)
Other *n* (%)		–	1 (1)
Currently on alternative anti-TNF *n* (%)		28 (43)	
Currently on other biologic agent *n* (%)		31 (48)	
Number of previously failed DMARDs *n* (%)			
0	8 (9)	7 (11)	39 (49)
1	27 (30)	15 (23)	24 (30)
2	26 (29)	10 (15)	8 (10)
3	15 (17)	14 (22)	6 (7)
>3	13 (15)	19 (29)	3 (4)

The most common information sources about prospective medications were rheumatology nurses (94%), rheumatology doctors (90%) and information leaflets (84%). Patients received information from internet/website, general practitioner, newspaper/TV, general practice nurse, other patients and friends/family less frequently. Information from rheumatology nurses, rheumatology doctors and information leaflets were found most useful. Internet/website information was significantly more useful in younger participants (*p* = 0.009). Patients with the longest disease duration found information from GP nurses (*p* = 0.011) and friends/family (*p* = 0.019) least useful. Participants in the failed anti-TNF group found information from general practitioner less useful (*p* = 0.006).

Participants were generally satisfied with the information received. Ninety-one percent said they received ‘almost enough’, ‘just right’ or ‘a bit too much’ information; 92% said the quality was ‘acceptable’, ‘good’ or ‘very good’; and 97% said the timing was ‘a bit too early’, ‘about right’ or ‘a bit too late’. When asked about how much information they had already known, 95% responded ‘none’, ‘a little’ or ‘some’. These responses varied little between verbal and written information.

Thirty-eight percent of respondents felt reassured/slightly reassured by information they received, 22% felt neutral, but 37% reported feeling more worried/slightly worried. The first anti-TNF group reported significantly greater reassurance than the other participants (*p* = 0.012).

Participants reported that rheumatology nurses were the easiest to ask when seeking further information, followed by rheumatology doctors and then hospital pharmacists. Health professionals in community settings (GPs, practice nurses, pharmacists) were felt less easy to ask, particularly for those with longer disease duration (*p* = 0.049, *p* = 0.002 and *p* = 0.019, respectively). Socioeconomic status had no apparent effect on information gathering from any source.

Exploring knowledge of common anti-TNF or DMARD side effects (Table 2), 40% of participants were aware of all important applicable side effects. Although, this was highest for the DMARD group (57%), excluding ‘theoretical increased risk from cancer’ from analysis, the anti-TNF groups performed at a similar level (55 and 62%, respectively).

**Table 2:** Knowledge of drug side effects by patent group

	Proportion who correctly identified that anti-TNF (first and failed anti-TNF groups) or participants’ current DMARDs (DMARD group) could cause different side effects			
Drug side effect	First anti-TNF group *n* (%)	Anti-TNF failure group *n* (%)	DMARD group *n* (%)	All relevant participants *n* (%)
Skin rash	56/97 (58)	57/86 (66)	14/24 (58)	127/ 207 (61)
Abnormal liver blood tests	–	–	59/79 (75)	59/79 (75)
Abnormal kidney blood tests	–	–	0/1 (0)	0/1 (0)
High blood pressure	–	–	8/11 (73)	8/11 (73)
Increased risk of infections	79/97 (81)	72/86 (84)	55/69 (80)	206/252 (82)
Theoretical increased risk from cancer	41/97 (42)	38/86 (44)	–	79/183 (43)
All correct	30/97 (31)	29/86 (34)	46/81 (57)	105/264 (40)

Participants’ concerns on starting new treatments were the highest with increased risk from cancer, increased infections and deteriorating mood. Older participants were reluctant to start medication potentially causing rashes (*p* ≤ 0.005) or limiting travel vaccinations (*p* = 0.007). DMARD patients were more reluctant to start medications across all potential side effect areas. The first anti-TNF patients were less concerned starting a medication potentially causing rashes (*p* = 0.049). The failed anti-TNF patients were less concerned about potential increased risk from cancer (*p* = 0.037). Female participants were more likely to continue medication with potential to increase risk from cancer (*p* = 0.038) or worsen mood (*p* = 0.033).

Participants were asked if anti-TNF/DMARD medication should be temporarily withheld if they experienced different symptoms/infections. They reported treatment cessation with chest infections (65%), vomiting (45%), urine infections (42%) and skin infections (42%). The failed anti-TNF group were most likely to believe medication should be withheld. Participants were asked whether they had actually stopped their medication during infections. Medication was continued in 778/1040 infective episodes (75%) despite patients believing they should have stopped in 139 of these cases (18%). Appropriate cessation occurred less in DMARD (31%) than that in the first anti-TNF (84%) and failed anti-TNF (78%) groups.

Attitudes, uptake and knowledge about vaccinations [22, 23] are shown in Table 3. Most participants (93%) felt annual influenza immunisation were important, with 76% of respondents being immunised annually. Seventeen percent were aware that influenza immunisation was important but only received it ‘some years’ or ‘never’. Amongst the anti-TNF groups, 13% believed that all vaccinations (including travel vaccinations) were safe while taking anti-TNF.

**Table 3:** Attitudes, uptake and knowledge about vaccinations

	First anti-TNF group %	Anti-TNF failure group %	DMARD group %	All participants %
Perceived importance of flu jab for patients with rheumatoid arthritis taking anti-TNF/DMARDs compared with ‘the man in the street’	(*N* = 94)	(*N* = 83)	(*N* = 77)	
Much less important	3	3	3	9
A bit less important	1	1	6	8
The same	7	4	8	19
A bit more important	13	13	15	41
Much more important	70	62	45	177
Reported uptake of flu jab	(*N* = 95)	(*N* = 84)	(*N* = 79)	
Never	16	13	17	46
Some years	8	6	2	16
Every year	71	65	60	196
Perceived safety of other vaccinations (for example travel vaccinations) for patients with rheumatoid arthritis taking anti-TNF/DMARDs	(*N* = 95)	(*N* = 84)	(*N* = 80)	
Safe	14	9	14	37
Not safe	25	37	21	83
Unsure	56	38	45	139

Attitudes, uptake and knowledge about cancer screening are shown in Table 4. Seventy-nine percent of participants believed that involvement in cancer screening programmes was important. Overall uptake of those eligible for cancer screening programmes were bowel cancer (stool sample) 89%, breast cancer (mammography) 94%, cervical cancer (smear) 87% and prostate cancer (PSA measurement) 43%. These proportions did not vary significantly with the patient group.

**Table 4:** Attitudes, uptake and knowledge about cancer screening

	First anti-TNF group *n* (%)	Anti-TNF failure group *n* (%)	DMARD group *n* (%)	All participants *n* (%)
Perceived importance of cancer screening for patients with RA taking anti-TNF/DMARDs compared with ‘the man in the street’	(*N* = 94)	(*N* = 82)	(*N* = 76)	(*N* = 252)
Much less important	0 (0)	0 (0)	0 (0)	0 (0)
A bit less important	20 (21)	12 (15)	20 (26)	52 (21)
The same	33 (35)	21 (26)	27 (36)	81 (32)
A bit more important	41 (44)	49 (60)	29 (38)	119 (47)
Much more important	0 (0)	0 (0)	0 (0)	0 (0)
Reported use of national cancer screening programmes (only individuals eligible based on age/gender included)				
Bowel cancer (stool sample)	22/27 (81)	24/28 (86)	24/24 (100)	70/79 (89)
Breast cancer (mammogram)	33/36 (92)	38/40 (95)	27/28 (96)	98/104 (94)
Cervical cancer (smear)	29/36 (81)	24/27 (89)	24/26 (92)	77/89 (87)
Prostate cancer (PSA)	12/25 (46)	7/21 (33)	11/24 (46)	30/70 (43)
Perceived need for anti-TNF/DMARD medication to be stopped if developed cancer	(*N* = 94)	(*N* = 84)	(*N* = 79)	(*N* = 257)
Never	4 (4)	2 (2)	2 (2)	8 (3)
Occasionally	0 (0)	2 (2)	1 (1)	3 (1)
Sometimes	6 (6)	7 (7)	8 (10)	21 (8)
Usually	9 (10)	12 (13)	1 (1)	22 (9)
Always	19 (20)	23 (27)	4 (5)	46 (18)
Do not know	56 (60)	38 (45)	63 (80)	157 (61)

Most participants reported feeling neutral/reassured by viral hepatitis (95%) and HIV (91%) testing. Most would agree to viral hepatitis (92%) or HIV (81%) testing. Younger participants were less concerned by the prospect of both tests (*p* = 0.043 and *p* ≤ 0.005), and more likely to agree to testing (*p* = 0.018 and *p* ≤ 0.005), as were male participants (*p* ≤ 0.005).

## Discussion

Study limitations include a moderate response rate (39%) affecting overall respondent numbers. Participants were predominantly white Caucasians, potentially limiting applicability of findings in other patient populations.

Participants found specialist sources (rheumatology nurses and doctors and information leaflets) most useful as additional information sources, particularly those initiating biologic therapies. Participants with longer disease duration found information from their friends/family and community nurses less useful, possibly indicating a preference for specialist rheumatology involvement. The DMARD group found information from rheumatology nurse teams less useful, in contrast to the anti-TNF group, who had closer monitoring with the dedicated rheumatology nurses. This highlights the need for identified case workers and a single point of contact regarding patient education.

Knowledge levels prior to medication counselling was poor, confirming patients’ need for education. Although participants highly rated the amount, quality and timing of information they received, education actually increased concern in similar numbers to those reporting reassurance. Different patients clearly require different levels of information when considering new medications, highlighting the importance of a patient-centred approach [24]. The first anti-TNF group participants were significantly more reassured than the anti-TNF failure group, who possibly have their viewpoint skewed by previous negative experiences.

Rheumatology nurses were preferential sources of information over other health professionals, supporting the integral role specialist nurses play in the rheumatology multidisciplinary team. There was also preference for other hospital specialists, particularly pronounced amongst patients on anti-TNF and with longer disease durations. This could represent increased patient dependency on specialist care, or that community health professionals are less comfortable providing opinions on more complex patients. The TNF groups reported easier access to specialists (particularly rheumatology doctors), probably due to closer specialist supervision.

Participant knowledge of drug adverse effects was lower than expected, with poor identification of side effects commonly associated with DMARDs/anti-TNF. This may be due to poor information recall, but still represents a shortfall in patient knowledge [4]. Participants in the anti-TNF groups were more accepting of potential side effects (including infections and risk from cancer). Possibly, patients with more severe disease accept higher risks to achieve symptomatic control [6]. Older participants were more accepting of potential cancer risks, but more concerned around risks of developing/worsening depression.

Attitudes towards temporarily withholding medications despite concurrent symptoms/infections varied depending on medication group. DMARD patients believed less often that medications should be stopped. It is concerning that the majority of patients did not withhold medications in presence of symptoms/infections, including those who were aware that they should. Although adherence was better in anti-TNF patients, the clinical risk is potentially considerable.

Vaccination knowledge was encouraging with vaccination uptake generally high, and most participants aware that annual influenza immunisation was important. Amongst the minority not receiving annual influenza immunisations, most still perceived it to be important. This discrepancy could relate to difficulty accessing immunisations or fear of injection/side effects. Although only a minority of respondents believed that all vaccinations were safe while taking anti-TNF, this group is important to identify as they could be at significant clinical risk, particularly if vaccination providers are unaware of RA medications (particularly hospital prescribed parenteral therapies).

Uptake of UK national cancer screening was good, with the exception of prostate cancer testing (no national register, patients not routinely invited to participate). Cancer screening is arguably particularly important for patients on anti-TNF medications, stopping these medications early should cancer develop potentially improving patient outcome. Current clinical practice could be improved if encouragement to engage with all cancer screening programmes was included in anti-TNF guidelines, particularly as most participants were unaware that anti-TNF would be stopped should they develop cancer.

There are high levels of patient acceptability for viral hepatitis and HIV testing, supporting the concept of increased testing advocated in other specialist settings [25]. Even in low HIV-prevalence populations, we would suggest including these tests for all prospective anti-TNF patients.

## Summary

Although drug counselling is a well-established part of clinical care, there is potential for further improvement to ensure that patients’ knowledge empowers them to act safely and appropriately. Current levels of patient education still result in potentially avoidable risks around vaccination, cancer screening and drug cessation for infection. Education by the rheumatology team, particularly specialist nurses, is highly valued by the majority of patients, particularly those with more complex disease.
